# A Genetic Screen Identifies a Critical Role for the WDR81‐WDR91 Complex in the Trafficking and Degradation of Tetherin

**DOI:** 10.1111/tra.12409

**Published:** 2016-05-25

**Authors:** Radu Rapiteanu, Luther J. Davis, James C. Williamson, Richard T. Timms, J. Paul Luzio, Paul J. Lehner

**Affiliations:** ^1^ Cambridge Institute for Medical ResearchUniversity of Cambridge School of Clinical Medicine, Wellcome Trust/MRC Building Biomedical CampusCambridgeCB2 0XYUK; ^2^ Departments of Medicine and Clinical BiochemistryUniversity of Cambridge School of Clinical Medicine, Wellcome Trust/MRC Building Biomedical CampusCambridgeCB2 0XYUK

**Keywords:** CAMRQ2, early endosomes, EGFR, endocytosis, HIV‐Vpu, human haploid cells, KBM7, lysosomes, tetherin, WDR81, WDR91

## Abstract

Tetherin (BST2/CD317) is a viral restriction factor that anchors enveloped viruses to host cells and limits viral spread. The HIV‐1 Vpu accessory protein counteracts tetherin by decreasing its cell surface expression and targeting it for ubiquitin‐dependent endolysosomal degradation. Although the Vpu‐mediated downregulation of tetherin has been extensively studied, the molecular details are not completely elucidated. We therefore used a forward genetic screen in human haploid KBM7 cells to identify novel genes required for tetherin trafficking. Our screen identified WDR81 as a novel gene required for tetherin trafficking and degradation in both the presence and absence of Vpu. WDR81 is a BEACH‐domain containing protein that is also required for the degradation of EGF‐stimulated epidermal growth factor receptor (EGFR) and functions in a complex with the WDR91 protein. In the absence of WDR81 the endolysosomal compartment appears swollen, with enlarged early and late endosomes and reduced delivery of endocytosed dextran to cathepsin‐active lysosomes. Our data suggest a role for the WDR81‐WDR91 complex in the fusion of endolysosomal compartments and the absence of WDR81 leads to impaired receptor trafficking and degradation.

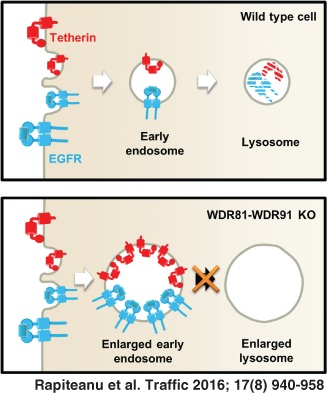

As obligate intracellular parasites, viruses have evolved multiple strategies to manipulate host cellular pathways, to enable their replication and evade immune detection. Conversely, mammalian cells have developed restriction factors that limit or prevent different stages of viral replication. The study of how viruses target host factors has provided key insights into our understanding of viral pathogenesis as well as fundamental aspects of host cell biology.

A good example of this is the HIV1‐Vpu mediated downregulation of the viral restriction factor, tetherin (BST2/CD317). Tetherin inhibits the release of a wide spectrum of enveloped viruses, including human immunodeficiency virus (HIV) and Ebola, from infected cells 1, 2, 3, 4, 5, 6, 7, 8, 9, 10, 11, 12, 13. Tetherin achieves this by targeting the lipid bilayer of the viral envelope, a molecular component that cannot be mutated or eliminated. Tetherin is an interferon inducible, transmembrane protein with an unusual topology, that is key to its antiviral function. It encodes an extended extra‐cellular alpha helix with both extremities embedded in cellular membranes using different anchors: an N‐terminal transmembrane domain and a C‐terminal glycosylphosphatidyl‐inositol (GPI) anchor 14, 15, 16, 17. This allows tetherin to form disulphide linked homodimers and anchor virions to cellular membranes through its terminal domains 17, 18, 19. Beyond limiting the spread of viruses, the tetherin‐mediated accumulation of virions at the plasma membrane (PM) enhances antibody opsonisation 20, 21, 22, 23, leads to virion endocytosis 1, 24 and can induce proinflammatory NF‐kB signalling 25, 26, 27. Tetherin localizes not only to PM lipid rafts but also to endocytic compartments and the trans‐Golgi network (TGN) 14. Its N‐terminal cytoplasmic tail contains the YDYCRV endocytic motif which binds the AP1 and AP2 clathrin adaptors and facilitates the endocytic recycling of tetherin via the TGN 28.

The HIV‐1 encoded accessory protein Vpu counteracts the inhibitory effects of tetherin, the HIV‐1 receptor CD4 and the amino acid transporter SNAT1. Vpu uses its DSGNES phosphodegron to recruit the same SCF^βTrCP2^ E3 ubiquitin ligase complex to facilitate the ubiquitination of tetherin 29, 30, 31, 32, CD4 33 and SNAT1 34, but targets the different proteins in distinct compartments and enhances their degradation via different mechanisms. Vpu traps newly synthesized CD4 in the ER and promotes its degradation through ER‐associated protein degradation (ERAD) 33, 35. In contrast to CD4 degradation, tetherin antagonism is abolished by artificial retention of Vpu in the ER 36 as Vpu targets tetherin in a post‐ER compartment 36. The tetherin–Vpu interaction occurs via their respective transmembrane domains and leads to a reduction of tetherin levels at the cell surface 19, 37, 38. Nevertheless, Vpu has little effect on the rate of PM endocytosis of tetherin 39, 40, 41 but rather interferes with the delivery of newly synthesized tetherin to the PM and with tetherin recycling via the TGN 39, 41. Consistent with the notion that Vpu prevents access of tetherin to the PM is the observation that both tetherin and Vpu colocalize mainly to TGN compartments in HIV‐infected cells 42, 43. Tetherin downregulation from the PM relies on clathrin‐mediated endocytosis 40, 43, Rab7 GTPase 44 and the endosomal sorting complex required for transport (ESCRT) proteins HRS 45, TSG101 45 and UBAP1 46.

Despite significant advances in our mechanistic understanding of Vpu‐mediated antagonism of tetherin, the molecular details are not fully elucidated 12, 47. For example, there is controversy surrounding Vpu's E_59_XXXLV_64_ motif. EXXXLV mutants can bind tetherin, the SCF^βTrCP2^ complex and HRS and yet have limited capability in decreasing cell surface expression or degradation of tetherin 48. Furthermore, both Vpu EXXXLV mutants and tetherin are present at the PM and both are incorporated in nascent virions 48. This suggested that Vpu's E_59_XXXLV_64_ motif might facilitate interaction with other, unknown components of the cellular machinery. Moreover, the requirement for SCF^βTrCP2^ in the Vpu‐mediated downregulation of PM tetherin has been much debated 30, 31, 37, 41, 49. A recent study addressed these issues and showed that SCF^βTrCP2^ is essential for the Vpu‐mediated degradation of tetherin but dispensable for tetherin downregulation from the PM in HIV‐1 infected cells 50. Furthermore, phosphorylation of Vpu's DSGNES motif serves not only for the recruitment of SCF^βTrCP2^, but is also required along with the intact E_59_XXXLV_64_ motif for binding to either clathrin adaptors AP‐1 or AP‐2 50, 51.

To further understand the molecular machinery required for Vpu‐mediated tetherin downregulation and degradation, we used a novel approach in the form of a forward genetic screen in Vpu‐expressing KBM7 cells. This methodology has proven successful in enabling the identification and characterization of novel genes required for viral entry 52 as well as intracellular viral evasion pathways 53, 54, 55. Our screen identified WDR81, a poorly characterized BEACH domain containing protein we find to be required for tetherin trafficking and degradation in both the presence and absence of Vpu. Upon WDR81 depletion, tetherin accumulates in enlarged endocytic vesicles/compartments. Furthermore, our data show that the loss of WDR81 delays the delivery of endocytosed fluid phase cargo to lysosomes and inhibits degradation of the EGFR. We find that WDR81 forms a complex with WDR91 and this interaction is critical for function. Therefore, by studying tetherin trafficking in mammalian cells, we have identified a WDR81‐WDR91 complex responsible for maintaining the morphology and function of the endolysosomal pathway.

## Results

### A haploid genetic screen identifies a requirement for WDR81 in the Vpu‐mediated downregulation of tetherin

**Figure 1:**
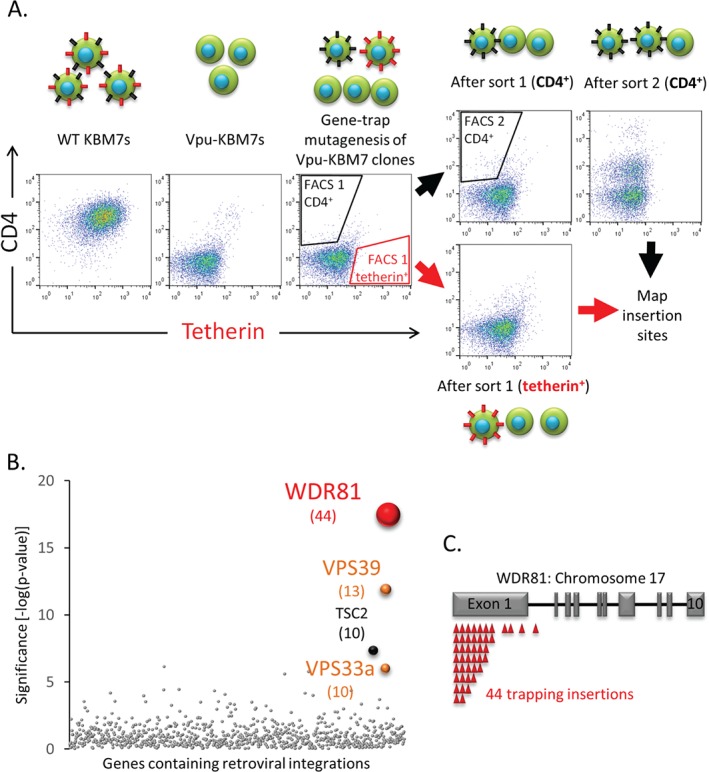
**A haploid genetic screen identifies a requirement for WDR81 in the Vpu‐mediated downregulation of tetherin.** A) Near‐haploid Vpu‐KBM7 cell clones were mutagenised with gene‐trap retroviruses and rare CD4^high^ cells selected by two sequential rounds of FACS. Rare tetherin^high^ cells were selected by one round of FACS. Approximate sorting gates are indicated. Schematic drawings of cells at various stages of the genetic screen are shown above or below respective scatterplots. Black spikes represent CD4, red spikes represent tetherin. B) Bubble plot illustrating the hits from the tetherin^high^ screen. Bubble size is proportional to the number of independent inactivating gene‐trap integrations identified (shown in brackets). C) Sequencing of retroviral insertion sites in the selected tetherin^high^ population revealed 44 independent inactivating insertions (red triangles) in the WDR81 gene on chromosome 17. Grey rectangles represent exons.

To identify novel host factors required for the HIV1‐Vpu‐mediated downregulation and degradation of tetherin and CD4, we performed a forward genetic screen in human near‐haploid KBM7 cells. We predicted that disruption of a gene essential for Vpu function would interfere with tetherin or CD4 downregulation and increase their cell surface expression, resulting in tetherin^high^ and/or CD4^high^ cells (Figure 1A). We created a cell line expressing HIV‐1 NL4.3 Vpu internal ribosome entry site (IRES) CFP (Vpu‐CFP) (Figure S1A, Supporting Information) and selected clones with low cell‐surface expression of endogenous tetherin and CD4. A total of three Vpu‐CFP clones were pooled and transduced with a gene‐trap retrovirus, at an MOI of 1 to prevent multiple retroviral gene‐trap mutations per cell. Seven days post‐mutagenesis, rare Vpu‐KBM7 cells that were either tetherin^high^CFP^high^ or CD4^high^CFP^high^ (Figure 1A) were selected by fluorescence‐activated cell sorting (FACS). The sorting gates were designed to avoid rare cells which have lost Vpu expression and thus became both tetherin^high^ and CD4^high^ (Figure S1B). Seven days after the first selection, the CD4^high^ population underwent a second FACS enrichment, which allowed visualization of a CD4^high^ tetherin^low^ cell population (Figure 1A), from which genomic DNA was extracted. The retroviral gene‐trap integration sites from this enriched CD4^high^ population were sequenced and mapped back to the genome resulting in the identification of three genes that were significantly enriched for inactivating insertions compared to an unselected control population 53: *βTrCP2 (FBXW11), CUL1* and *UBE2M* (Figure S1C). The discovery of these genes validated our genetic approach as they are all members of the SCF^βTrCP2^ E3 ubiquitin ligase complex, known to be recruited by Vpu to facilitate the ubiquitination and subsequent degradation of CD4 29, 30, 31, 32, 33. Although depletion of βTrCP2 in Vpu‐expressing KBM7 cells (Figure S1D) restores PM levels of CD4, cell surface expression of tetherin is not rescued, explaining why component genes of the SCF^βTrCP2^ complex were not identified as hits in the tetherin^high^ screen. These findings are in agreement with a recent study that found βTrCP2 to be dispensable for the Vpu‐mediated downregulation of tetherin from the PM 50. We observed the same effect, with a full rescue of PM CD4 levels but no restoration of cell surface tetherin, when depleting βTrCP2 in Vpu‐expressing CEMT4 cells, a human leukemic CD4+ T‐cell line used in HIV studies (Figure S1E). CEMT4 cells appear more susceptible to CRISPR‐Cas9‐mediated editing, showing better knock‐out (KO) efficiency than KBM7 cells (compare Figure S1D with E and data not shown) and were thus used for the validation of further hits.

A second FACS enrichment on the tetherin^high^ population was unsuccessful as following selection the majority of the sorted cells died. Genomic DNA was therefore extracted from cells recovered from the first tetherin^high^ sort (Figure 1A). Mapping of these gene‐trap integration sites identified four genes: *VPS33a*, *VPS39*, *TSC2* and *WDR81* (Figure 1B). VPS33a and VPS39 are known components of the homotypic fusion and vacuole protein sorting complex (HOPS) 56, 57, 58. The HOPS complex is essential for endosomal maturation 59, delivery of cargo to lysosomes 60, 61 and fusion of autophagosomes with late endosomes/lysosomes 62. TSC2 is a negative regulator of the mTORC1 complex [reviewed in 63] and has also been described as a Rab5 GTPase activating protein (GAP) 64, 65. We chose to focus on the top hit, *WDR81,* a large and poorly characterized gene which had 44 independent trapping integrations in transcript 1 (ENST00000409644) (Figure 1C).

**Figure 2:**
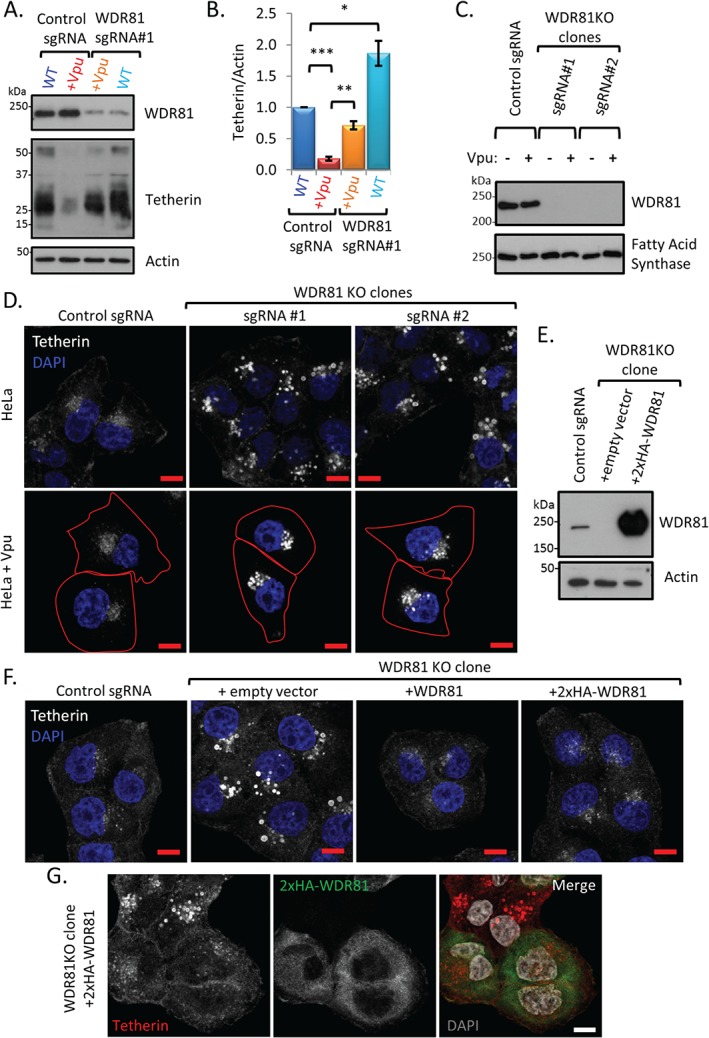
**Depletion of WDR81 results in accumulation of tetherin in vesicular compartments in both the presence and absence of Vpu.** A) WDR81 and tetherin expression was assessed by immunoblotting control and WDR81 depleted CEMT4 cells ± Vpu. B) Tetherin levels from three independent experiments using cells in ‘A’ were quantified with Image J and normalized by loading. Histograms represent mean normalized tetherin signal ±SEM. *p ≤ 0.05, **p ≤ 0.01, ***p ≤ 0.001. C) WDR81 expression was assessed by immunoblot in HeLa cell clones ± Vpu stably expressing Cas9 and two independent WDR81 specific sgRNAs. D) Confocal microscopy images of endogenous tetherin using cells in ‘C’. Cell edges in Vpu expressing cells are outlined in red. E) HeLa cells were depleted of WDR81 by transient expression of Cas9 and WDR81 sgRNA#1 and single cell cloned. Exogenous expression of 2×HA‐WDR81 in these WDR81KO clones was analysed by immunoblot. F) Immunostaining of endogenous tetherin in control, WDR81KO clones and WDR81KO clones genetically complemented with exogenous WDR81 or 2×HA‐WDR81. G) Immunostaining of tetherin and 2HA‐WDR81 in genetically complemented WDR81KO cells (prior to blasticidin selection). All scale bars represent 10 µm.

To verify the requirement for WDR81 in the Vpu‐mediated downregulation of tetherin, we used the CRISPR‐Cas9 system to KO WDR81 in CEMT4 cells. We confirmed depletion of WDR81 in the sgRNA‐targeted CEMT4 cell population, as the predicted 210‐kDa WDR81 band detected in control CEMT4 cells was markedly reduced in the WDR81KO mixed cell population (Figure 2A). Immunoblot analysis showed that the Vpu‐mediated decrease in tetherin levels seen in Vpu‐CEMT4 cells (Figure 2A, lane 2 and three replicates quantified in B) was rescued following depletion of WDR81 (Figure 2A, lane 3 and three replicates quantified in B). Furthermore, even in the absence of Vpu, depletion of WDR81 caused a significant increase in tetherin levels (Figure 2A, lane 4, and three replicates quantified in B). Interestingly, the marked increase in the intracellular tetherin pool resulted in only a modest rescue (1.6‐fold) of cell surface tetherin in WDR81‐depleted, Vpu‐CEMT4s (Figure S2A and three replicates quantified in B), suggesting that accumulation of cellular tetherin occurs predominantly in an intracellular compartment.

To identify where tetherin accumulates in the absence of WDR81, we generated HeLa cell clones deficient in WDR81 (HeLa‐WDR81KO) ± Vpu. Two independent sgRNAs targeting WDR81 exon 4 and 5 generated WDR81 deficient clones as confirmed using immunoblot analysis (Figure 2C). Vpu was stably expressed in WDR81 knockout clones and control HeLa cells and, as expected, both flow cytometry and confocal immunofluorescence microscopy confirmed that both the cell surface and the intracellular pool of tetherin was reduced in Vpu‐expressing HeLa cells (Figure S2C and D). In the two WDR81‐knockout clones, tetherin accumulated in swollen, perinuclear, vesicular compartments in both the presence and absence of Vpu (Figure 2D). To confirm this observation and exclude possible artefacts associated with stable Cas9 and sgRNA expression, we carried out a rescue experiment. First, we created a further WDR81KO HeLa cell line by transient expression of Cas9 and WDR81‐sgRNA #1 (targeting WDR81 exon 5). Single cell clones were selected and loss of WDR81 again confirmed (Figure 2E). The intracellular accumulation of tetherin seen in the WDR81KO clone was rescued by stable expression of WDR81 [untagged or N‐terminus 2×HA tagged (Figure 2E)], confirming that loss of WDR81 was responsible for the observed phenotype (Figure 2F). Furthermore, the accumulation of tetherin in swollen vesicular compartments was only seen in those cells not complemented by 2×HA‐WDR81 (Figure 2G). Thus, we validated WDR81 as required for tetherin trafficking and degradation.

### WDR81 is a BEACH domain containing protein that associates with endolysosomal membranes

**Figure 3:**
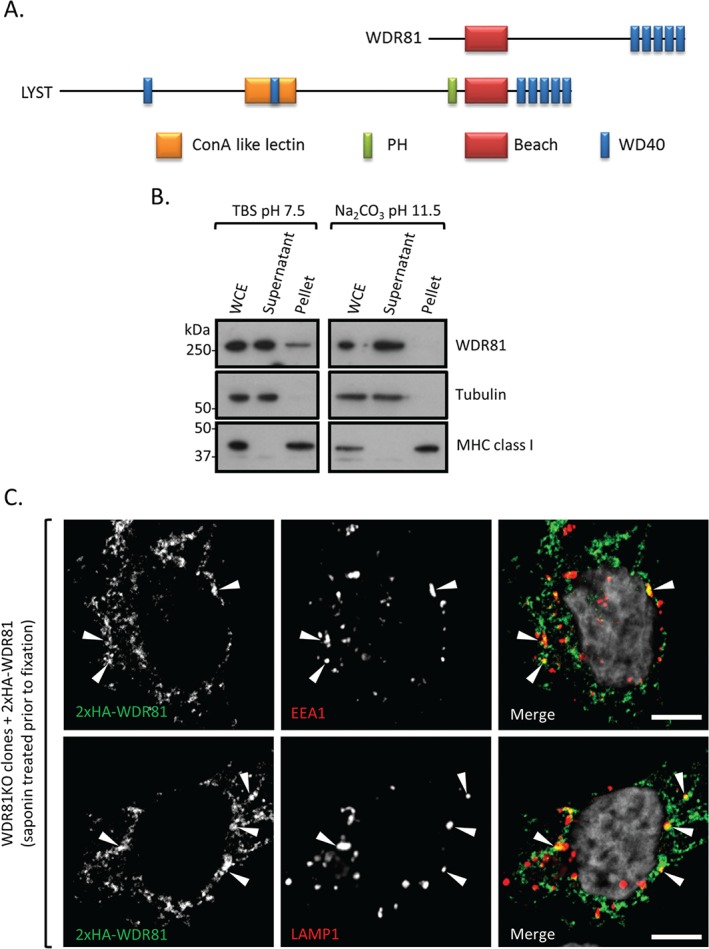
**WDR81 is a BEACH domain containing protein that associates with endolysosomal membranes.** A) Schematic view of WDR81 and LYST protein domains. B) Ultracentrifugation of HeLa cell homogenates in neutral pH TBS buffer and highly basic sodium carbonate buffer. WCE, whole cell extract. C) Immunostaining of 2×HA‐WDR81 and EEA1 or LAMP1 in cytosol extracted HeLa cells. WDR81KO cells complemented with exogenous 2xHA‐WDR81 were permeabilized with saponin to extract non‐membrane‐associated proteins and fixed before immunolabelling. Scale bars: 5 µm.

WDR81 is a 1941 amino acid, Beach (after ‘Beige and Chediak–Higashi’) domain containing protein (BDCP) with five C‐terminal WD40 domains (Figure 3A). Of the nine known human BDCPs, LYST is the best studied and is mutated in Chediak–Higashi syndrome (CHS) 66, 67. BDCPs are cytoplasmic, membrane‐associated proteins known to function in endolysosomal trafficking [reviewed in 68]. Although initially described as a transmembrane, mitochondrial protein 69, 70, TMHMM2.0 analysis of WDR81 showed no characteristic features of an integral membrane protein 71, and ultracentrifugation of cell homogenates did not support this assertion (Figure 3B). Indeed, ultracentrifugation of cell homogenates showed that WDR81 was predominantly found in the soluble fraction at neutral pH and entirely in the soluble fraction at high pH (Figure 3B). This suggests that WDR81 is a cytosolic protein which may associate with membranes.

We next sought to identify the compartment(s) with which WDR81 associates. Exogenous expression of 2×HA‐WDR81 in WDR81KO cells rescues the accumulation of tetherin from enlarged perinuclear vesicles (Figure 2F and G) but leads to significantly higher protein levels as compared to wild‐type cells (Figure 2E) which limit sub‐cellular localization studies. Extraction of non‐membrane‐associated proteins by a brief saponin wash prior to fixation allowed us to image the membrane bound fraction of 2×HA‐WDR81. We found that membrane‐associated 2×HA‐WDR81 partially colocalizes with both early EEA1 positive and late LAMP1 positive endocytic compartments (Figure 3C). The remaining WDR81 pool observed in Figure 3C probably reflects remaining cytosolic protein due to incomplete extraction and/or other membrane‐associated protein. Nevertheless, taken together, Figures 3B and C suggest that WDR81 associates with endolysosomal membranes.

### WDR81 modulates endolysosomal morphology and the delivery of endocytosed cargo to late endo‐lysosomal compartments

**Figure 4:**
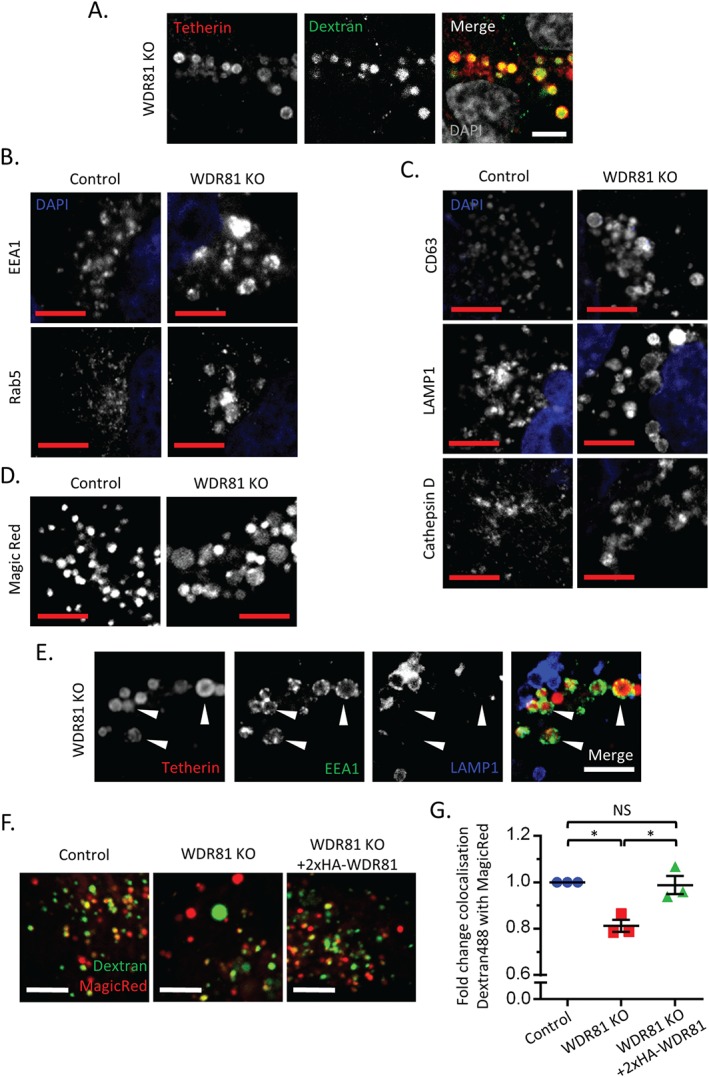
**WDR81 depletion alters endolysosomal morphology and trafficking, and promotes the accumulation of tetherin in swollen EEA1 positive vesicles.** A) Confocal immunostaining of tetherin (red) in WDR81KO HeLa cells loaded with dextran Alexa Fluor 488 (green) for 16 h. B–C) Confocal microscopy images of early (B) and late (C) endolysosomal markers in control and WDR81KO HeLa cells. D) Confocal live microscopy of control and WDR81KO cells incubated with Magic Red. E) Confocal immunostaining of tetherin (red), EEA1 (green) and LAMP1 (blue) in WDR81 KO cells. F) Representative live‐cell confocal microscopy images of control, WDR81 and 2×HA‐WDR81 complemented WDR81KO HeLa cells that had been loaded with dextran Alexa Fluor 488 (green) for 2 h, chased for 1 h and then stained with Magic Red (red). G) Quantification of colocalization of dextran Alexa Fluor 488 with Magic Red using images captured by live‐cell confocal microscopy of the cells in ‘F’. Mean ± SEM of three independent experiments with five fields each, ≥30 cells total per condition. *p ≤ 0.05. All scale bars represent 5 µm.

Because tetherin traffics through the endolysosomal pathway, we wanted to identify the site of tetherin accumulation in the absence of WDR81. Our prediction that the swollen tetherin positive vesicles seen in WDR81 knockout cells were enlarged endosomal and/or lysosomal compartments was confirmed by the accessibility of these vesicles to endocytosed, fluid phase dextran (Figure 4A). To further characterize these swollen endolysosomal compartments in WDR81 depleted cells we examined the distribution of known endolysosomal markers. Confocal microscopy images showed that swollen compartments were positive for early endosomal markers (EEA1 and Rab5) (Figure 4B) and late endosomal/lysosomal markers (CD63, LAMP1 and Cathepsin D) (Figure 4C). Thus, WDR81 is required for maintaining the correct morphology of both early and late endocytic compartments.

Our findings of impaired tetherin degradation, together with the accumulation of tetherin in swollen endolysosomal compartments in the absence of WDR81 (Figure 1D) questioned whether the loss of WDR81 affected lysosomal acidification or trafficking of lysosomal proteases such that the degradative capacity of the lysosomal compartment is impaired. Live‐cell confocal microscopy of LysoSensor Yellow/Blue DND‐160 labelled WDR81KO cells showed that some of the swollen compartments were acidic (Figure S3A and quantified in B). The presence of both neutral and acidic swollen compartments is consistent with the idea that depletion of WDR81 causes the enlargement of both early (neutral) and late (acidic) endosomes. Quantification of yellow/blue ratios revealed that depletion of WDR81 causes higher acidification of late endosomal compartments (Figure S3B). Furthermore, swollen compartments were readily labelled by the membrane permeable, cresyl violet‐conjugated Magic Red substrate for Cathepsin B (Figure 4D). Together these experiments suggest that in the absence of WDR81, these swollen endolysosomal compartments are both acidified and contain functional lysosomal proteases.

Because the degradative capacity of late endolysosomal compartments appeared unimpaired in WDR81‐depleted cells, we predicted that tetherin accumulation should occur in early compartments. To test this, we fluorescently labelled both control and WDR81KO cells for tetherin together with the early endosomal marker EEA1 and/or the late endosomal/lysosomal marker LAMP1. In wild‐type HeLa cells, the intracellular pool of tetherin localizes predominantly within EEA1 positive vesicles [Figure S3C and 45] but not within LAMP1 positive vesicles (Figure S3D). In the absence of WDR81, the early EEA1 positive and late LAMP1 positive compartments are distinct and tetherin accumulation is predominantly seen within early, swollen EEA1 positive vesicles (Figure 4E and S3C) but not within late, swollen LAMP1 positive vesicles (Figure 4E and S3D).

Taken together, the data in Figures 2D and 4B–E suggest that WDR81 depletion abolishes maturation of endosomal compartments and/or impairs the delivery of cargo from early to late endolysosomal compartments. To examine this further we determined how loss of WDR81 affected the delivery of endocytosed fluorescent dextran to Magic Red‐positive, cathepsin‐active organelles using a published assay, previously used to show the requirement for mammalian homologs of the HOPS complex in delivery of endocytosed dextran to cathepsin‐active lysosomes 60, 61. Using the control, WDR81KO and rescued (2×HA‐WDR81 complemented) HeLa cell lines described in Figures 2E and F, we observed a reproducible reduction in delivery of dextran to Magic Red‐positive organelles in WDR81KO HeLa cells, that was clearly rescued by expression of WDR81 cDNA (Figure 4F and G). These data are consistent with a requirement for WDR81 in the maturation of endosomal compartments and/or an impairment in the delivery of cargo to late endolysosomal compartments.

### WDR81 depletion inhibits the degradation of EGFR by sequestering it in early endosomal compartments

**Figure 5:**
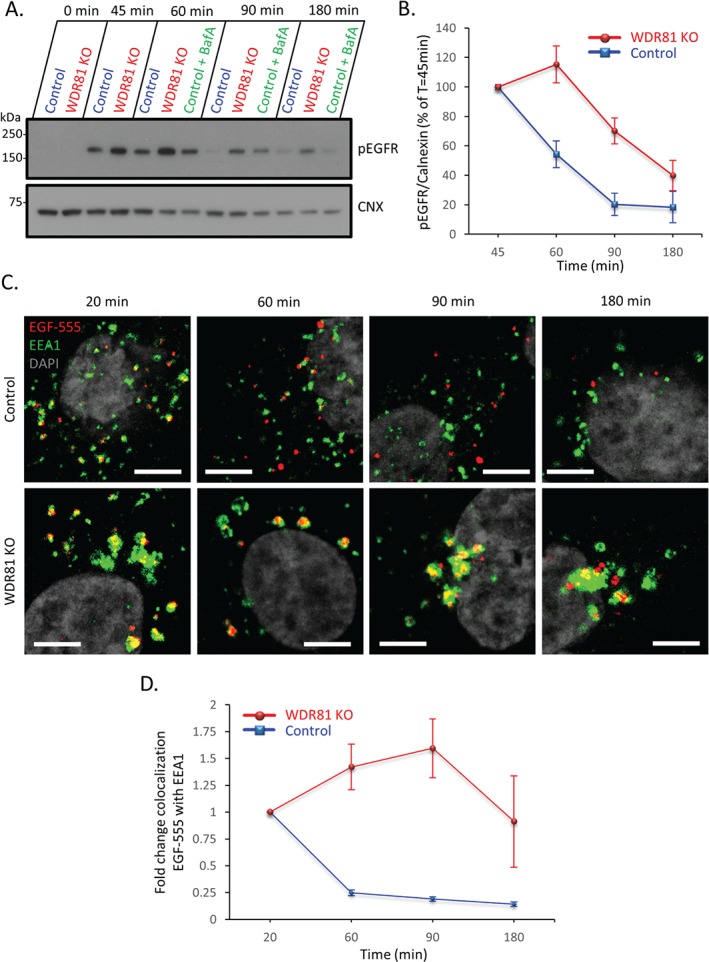
**WDR81 depletion delays EGFR degradation and promotes EGFR accumulation in early EEA1 positive endosomal compartments.** A) Control, WDR81KO and bafilomycin A (400 nm) treated control cells were stimulated with EGF for the indicated times and analysed using immunoblot for phosphorylated EGFR (pEGFR). CNX, calnexin. B) pEGFR signal from three replicates of the experiment shown in ‘A’ was quantified using Image J, normalized by loading and the average normalized pEGFR levels from control and WDR81KO cells were plotted to reflect percentage of normalized pEGFR at 45 min ±SEM. C) Confocal microscopy of control and WDR81KO HeLa cells stimulated with EGF‐Alexa‐555 for 5 min (red) and chased for the indicated times. Cells were then fixed and stained for EEA1 (green). Scale bars represent 10 µm. D) Quantification of colocalization (normalized to the 20 min time point) of EGF‐Alexa‐555 with EEA1 using images captured by live‐cell confocal microscopy of the cells in ‘C’. Mean ± SEM of three independent experiments with ≥5 fields (average two cells per field) each per condition. Scale bars represent 5 µm.

Because the depletion of WDR81 affected endolysosomal maturation and the delivery of tetherin to late endolysosomes, we wanted to determine whether WDR81 depletion also affects the degradation of other cell surface proteins. We therefore examined the trafficking of the well‐characterized EGFR, which is phosphorylated upon EGF ligand binding, rapidly endocytosed and trafficked to lysosomes. The degradation of endocytosed EGFR was examined in control and WDR81KO HeLa cells by pulsing cells with EGF, chasing for the specified times and immunoblotting solubilized cells for phosphorylated EGFR (pEGFR) (Figure 5A). In WDR81‐depleted cells we observed a delay in the degradation of pEGFR (Figure 5B). In the absence of WDR81, delayed EGFR degradation was also observed using confocal microscopy of EGF‐Alexa 555 pulsed cells (Figure S4).

Having shown that in WDR81KO cells tetherin accumulates in early EEA1 positive endosomal compartments (Figure 4E and S3C), we next asked whether the delay in EGFR degradation seen in WDR81KO cells (Figure 5A and B) is due to EGFR accumulation in EEA1 positive endosomes. An accumulation of EGF‐Alexa‐555 bound EGFR in EEA1 positive compartments was seen in WDR81‐depleted cells (Figure 5C and D), consistent with a defect in exit from EEA1 positive endosomes. Taken together, Figure 4E, S3C, 5C and D suggest that depletion of WDR81 delays exit of endocytosed cargo from early endosomal compartments.

### WDR81 function is dependent on interaction with WDR91

**Figure 6:**
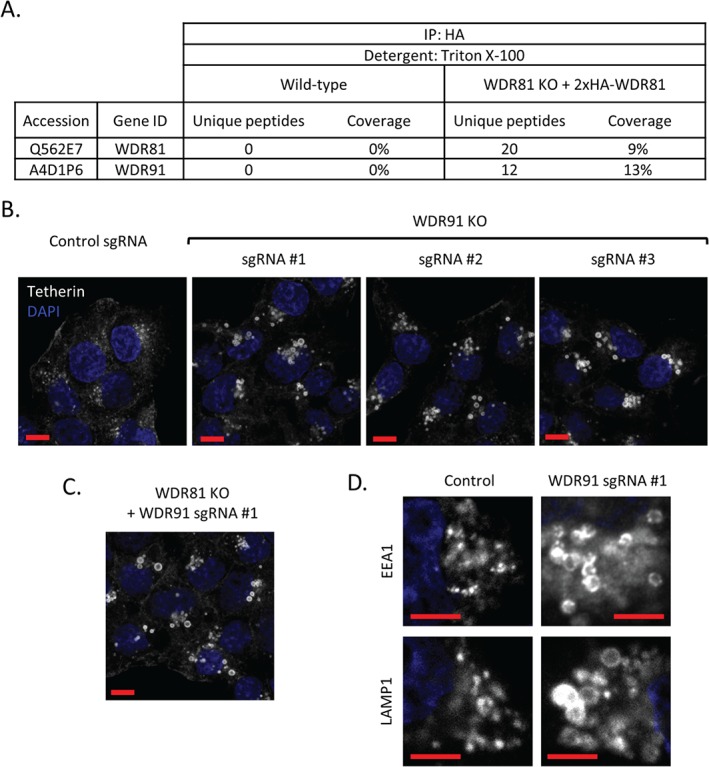
**WDR81 forms a functional complex with WDR91.** A) Mass spectrometry analysis of 2×HA‐WDR81 immunoprecipitates from 2×HA‐WDR81 expressing HeLa cells as compared with wild‐type control HeLa cells identifies WDR91 as a WDR81 binding partner. B) WDR91 was depleted in HeLa cells by stable expression of Cas9 and three independent WDR91‐specific sgRNAs. Tetherin trafficking in the resulting WDR91 KO cell lines and controls was analysed using confocal immunostaining. Scale bars represent 10 µm. C) WDR91 was depleted in WDR81KO clones and tetherin distribution was imaged using confocal microscopy. Scale bars represent 10 µm. D) Confocal microscopy images of early EEA1 positive and late LAMP1 positive endolysosomal compartments in control and WDR91 depleted HeLa cells. Scale bars represent 5 µm.

To determine if WDR81 acts alone, or in complex with other proteins, we used immunoprecipitation and mass spectrometry to identify binding partners of WDR81. Immunoprecipitation of HA‐tagged WDR81 from WDR81 knockout HeLa cells complemented with 2×HA‐WDR81 was performed. After subtracting proteins also present in control immunoprecipitates, WDR91 was identified as a WDR81 interaction partner (with 12 unique peptides, Figure 6A). WDR91 is a 747 amino acid protein whose bioinformatic analysis revealed 7 C‐terminal WD40 domains. To determine whether the interaction between WDR81 and WDR91 was of functional importance we used the CRISPR‐Cas9 system and three WDR91 sgRNAs to generate independent WDR91 deficient cell lines. In each of these cell lines, we observed accumulation of tetherin in swollen endolysosomes, identical to those seen following depletion of WDR81 (Figure 6B). The depletion of WDR81 together with WDR91 did not exacerbate tetherin accumulation (Figure 6C). Furthermore, we observed enlargement of both early EEA1 positive and late LAMP1 positive endolysosomal compartments in WDR91 depleted cells (Figure 6D), as seen in WDR81KO cells. Interestingly, a C‐terminal GFP fusion, which rendered an exogenous WDR81 construct non‐functional (i.e. unable to rescue tetherin accumulation in WDR81KO cells, Figure S5A), was unable to bind WDR91 (Figure S5B). Taken together, the data shown in Figures 6 and S5 suggest that interaction of WDR81 and WDR91 is critical for function.

## Discussion

In this study, our forward genetic screen identified a requirement for WDR81 in the HIV‐Vpu‐mediated degradation of tetherin. Subsequent experiments showed that WDR81 may play a more general role in the degradation of internalized PM proteins, that is independent of HIV‐Vpu. The degradation of both tetherin and pEGFR was delayed in WDR81‐depleted cells with both tetherin and EGFR accumulating in enlarged, early, EEA1 positive endosomes. WDR81 associates with both early and late endolysosomal compartments and the loss of WDR81 affected the morphology of the endolysosomal pathway with the appearance of swollen early and late compartments. This was accompanied by impaired delivery of fluid phase dextran cargo to cathepsin‐active lysosomes. Nevertheless, lysosomes of WDR81‐depleted cells were able to acidify and contained functional lysosomal proteases. We also found that WDR81 forms a complex with WDR91, which is required for correct function of these proteins. Our data suggest that the WDR81‐WDR91 complex is required for the trafficking and degradation of internalized PM proteins and facilitates delivery of cargo from early to late endosomal/lysosomal compartments. Our findings are consistent with a recent report from Liu et al. 72, published during preparation of our manuscript, which identified the *Caenorhabditis elegans* orthologues of WDR81 and 91 (respectively F25C9.1 or SORF‐2 and ZK563.5 or SORF‐1), as genes required for partial rescue of the endosome/lysosome fusion defects present in coelomocytes of *C. elegans vps18* mutants and showed that mammalian WDR81 and WDR91 form a complex functioning in endolysosomal trafficking.

The development of forward genetic screens in haploid human cells 73 provides a powerful approach to dissect cellular pathways in mammalian systems. Initially used in lethality screens 52, 73, 74, this approach has more recently been applied to the selection of either cell surface or intracellular phenotypes 53, 54, 55, 75, and was used here to identify components of the endocytic machinery. To effectively enrich for the desired cellular phenotype may require at least two FACS selections and is therefore dependent on preserving cell viability throughout the timescale of the selection process, which may be up to 28 days. HIV‐Vpu degrades CD4 from the early secretory pathway via the cellular ERAD machinery, while degradation of tetherin and SNAT1 occurs in the late secretory pathway. Our genetic approach was successful in identifying critical host cell components, including the E3 ligase βTrCP2, required for Vpu‐mediated CD4 downregulation, but the identification of cellular genes required for Vpu‐mediated downregulation of tetherin proved more challenging as the cells only survived a single round of positive FACS‐selection. The reasons for this are not entirely clear, but suggest that cells may be less tolerant of perturbations in the late as compared with the early secretory pathway.

Despite the selection for tetherin^high^‐expressing cells only surviving a single enrichment, our screen identified four significant genes which function in the endocytic pathway (Figure 1B). VPS33a and VPS39 are components of the HOPS complex 56, 57, 58, which function in endosomal maturation 59, delivery of cargo to lysosomes 60, 61 and fusion of autophagosomes with late endosomes/lysosomes 62, and are therefore promising candidates for interference with tetherin downregulation and degradation. Furthermore, siRNA‐mediated depletion of the HOPS components VPS33a 76 and VPS41 77, 78 impairs degradation of EGFR.

We focussed on the top hit of our screen, as WDR81 is a poorly characterized protein and one of nine BEACH domain containing proteins (BDCP) [reviewed in 68]. The BEACH domain was initially identified as a conserved region within LYST but its exact function remains unclear. Unlike other BDCPs which have a C‐terminus proximal BEACH domain preceded by a pleckstrin homology (PH) domain, the BEACH domain of WDR81 is close to the N‐terminus with no predicted PH domain (Figure 3A). As its name implies, WDR81 has multiple WD40 repeat (WDR) domains known to form scaffolds on which large protein complexes assemble [reviewed in 79, 80]. Although WDR81 was earlier suggested being an integral membrane spanning protein 69, both our bioinformatic and biochemical (Figure 3B) analysis suggest that WDR81 is cytosolic and it associates with EEA1 positive and LAMP1 positive endosomal membranes. This is in agreement with the recent findings of Liu et al. 72 who showed the association/colocalization of WDR81 with EEA1‐ and Rab7‐positive endosomes.

The depletion of WDR81 led to only a modest increase in cell surface tetherin, but a marked accumulation of intracellular tetherin that was apparent in both Vpu‐sufficient and Vpu‐deficient cells. Taken together, our results suggest that this is due to a perturbation in delivery of endocytosed cargo to lysosomes caused by accumulation of cargo in early endosomal compartments. The recent findings of Liu et al. 72 provide important mechanistic insight into our own observations on the defect of tetherin trafficking and degradation seen in WDR81‐ and WDR91‐depleted cells. They showed that WDR81 and WDR91 were able to regulate phosphatidylinositol 3‐phosphate (PtdIns3P) levels on endosomal membranes. Consequently, depletion of WDR81 and WDR91 led to the accumulation of PtdIns3P on early endosomal membranes, which caused excessive fusion of early endosomes and delayed early‐to‐late endosome conversion 72. Indeed, delayed trafficking of endocytosed fluid phase markers and altered endolysosomal morphology with enlarged early and late endolysosomal compartments was observed in WDR81‐depleted mammalian cells [present study and 72] as well as in F25C9.1 (WDR81 ortholog) depleted *C. elegans* coelomocytes, suggesting that this phenotype is evolutionarily conserved from *C. elegans* to humans. Although Liu et al. also observed that the WDR81‐WDR91 complex interacts with Beclin1 to inhibit the phosphatidylinositol 3‐kinase complex 72, we did not detect Beclin1 in our WDR81 mass spec IP suggesting that this interaction might be relatively weak.

Mutations in several BDCPs cause human disease [reviewed in 68] and WDR81 is no exception. The P856L mutation in WDR81 is associated with cerebellar ataxia, mental retardation, and disequilibrium syndrome 2 (CAMRQ2) 69. It would therefore be interesting to determine whether this mutation causes the same phenotype as WDR81 depletion or whether P856L acts as a gain of function mutation. Because the mutation occurs in the central region of WDR81, further studies should aim to identify whether it abolishes binding to WDR91.

In summary, we have shown a critical role for the WDR81‐WDR91 complex in facilitating endosomal maturation and delivery of cargo to late endolysosomes. Furthermore, our results provide evidence for the role of the WDR81‐WDR91 complex in facilitating the downregulation and subsequent degradation of cell surface receptors.

## Materials and Methods

### Plasmids

Codon optimized NL4‐3 Vpu was subcloned from pCR3.1‐Vpu‐HA 38 into the lentiviral expression vector pHRSIN‐Rox‐P_SFFV_‐GFP‐P_PGK_‐puromycin^R^‐Rox 54 in place of the GFP. The resulting plasmid was further modified downstream of Vpu with an internal ribosome entry site (IRES) CFP to generate the pHRSIN‐Rox‐P_SFFV_‐Vpu‐IRES‐CFP‐P_PGK_‐puromycin^R^‐Rox expression vector.

C terminally GFP tagged WDR81 was subcloned from the pCS2‐WDR81‐GFP construct (Creative Biogene) into the pHRSIN‐P_PGK_‐blasticidin^R^ background to generate pHRSIN‐P_SFFV_‐WDR81‐GFP‐P_PGK_‐blasticidin^R^. The construct was further modified to accommodate a double HA tag at the N terminus of WDR81 thus generating pHRSIN‐P_SFFV_‐2xHA‐WDR81‐P_PGK_‐blasticidin^R^.

*Streptococcus pyogenes* Cas9 was subcloned from the PX458 plasmid [Addgene; created by Feng Zhang, Massachusetts Institute of Technology, Boston, MA 81] into a pHRSIN backbone creating pHRSIN‐P_SFFV_‐FLAG‐NLS‐Cas9‐NLS‐P_PGK_‐hygromycin^R^. Short guide RNAs (sgRNA) were expressed from the pKLV‐U6gRNA(BbsI)‐PGKpuro2ABFP plasmid [Addgene; created by Kosuke Yusa, Wellcome Trust Sanger Institute, Hinxton, Cambridge, UK 82] targeting WDR81 and WDR91, respectively. Specific sgRNA sequences used: WDR81 sgRNA#1 5′‐AGGTGGCCACGGGCTCGCTC‐3′ (exon 5); WDR81 sgRNA#2 5′‐CCTCATGGCTGATCCGGGGC‐3′ (exon 4); WDR91 sgRNA#1 5′‐CAGCTCGTCAGTGCGCTCCA‐3′ (exon 1); WDR91 sgRNA#2 5′‐GTAGCTCCAATAATCCCGAA‐3′ (exon 2); WDR91 sgRNA#3 5′‐GTCTGTATATATCCTCCAAG‐3′ (exon 2); All plasmids were sequenced by Source Bioscience.

### Cell lines

KBM7, CEMT4 and HeLa cells were maintained in IMDM supplemented with 10% foetal calf serum and penicillin/streptomycin. Stable cell lines were produced by lentivirus transduction. Lentivirus was produced by cotransfecting 293ET cells with lentiviral vectors along with the packaging plasmids pCMVΔR8.91 and pMD.G using TransIT‐293 (Mirus) according to the manufacturer's instructions. Viral supernatant was harvested 48 h post‐transfection, filtered and transferred to cells, followed by spin infection at 1800 rpm for 45 min.

### Antibodies

Mouse anti‐Tetherin (APC coupled, BioLegend, 348410) and mouse anti‐CD4 (PE coupled, BioLegend, 300508) were used for flow‐cytometry and FACS. Antibodies used for immunoblotting include: rabbit anti‐WDR81 (Abcam, ab1217333, LOT GR98370‐3); rabbit anti‐Tetherin [NIH AIDS Reagent Programme, 11721, LOT 110114, 83]; rabbit anti‐Phospho‐EGF Receptor (Cell Signalling, 2234); mouse anti‐MHC‐I heavy chain (clone 3B10.7); mouse anti‐Fatty Acid Synthase (BD, 610962); mouse anti‐α‐tubulin (Sigma, T9026); mouse anti‐ß‐actin (Sigma, A2228); mouse anti‐calnexin (BD, 610962); goat anti‐mouse‐HRP (Jackson ImmunoResearch, 115‐035‐062) and goat anti‐rabbit‐HRP (Jackson ImmunoResearch, 111‐035‐144). Antibodies used for confocal microscopy: rabbit anti‐Tetherin [NIH AIDS Reagent Programme, 11721, LOT 110114, 83]; mouse anti‐HA (BioLegend, 901502); mouse anti‐EEA1 (BD, 610457); mouse anti‐Rab5 (BD, 610724); mouse anti‐CD63 (BD, 556019); mouse anti‐LAMP1 (BD, 555798); mouse anti‐LAMP1 (Alexa Fluor 647 conjugated, BD, 562622); rabbit anti‐Cathepsin D (Millipore, 219361); donkey anti‐rabbit IgG (Alexa Fluor 488 conjugate, Thermo Fisher Scientific, A‐21206); donkey anti‐mouse IgG (Alexa Fluor 647 conjugate, Thermo Fisher Scientific, A‐31571); goat anti‐mouse IgG (Pacific Blue conjugate, Thermo Fisher Scientific, P31582).

### Haploid genetic screen

The screen followed previously described protocols 53 with a number of adjustments. Firstly, to avoid potential artefacts associated with single proviral integration sites, we pooled three Vpu‐IRES‐CFP KBM7 clones that express low PM levels of both Tetherin and CD4 and expanded them to 100 million cells prior to mutagenesis. The gene‐trap vector used resembles the previously described pGT‐GFP‐pA 75, with the exception of a loxP cassette which was subcloned into the 3′ LTR. One week post‐mutagenesis, cells were selected for either Tetherin^high^ or CD4^high^. The collected CD4^high^ population was subjected to a second enrichment prior to genomic DNA extraction. For the tetherin^high^ screen, genomic DNA was extracted from the cells recovered after the first sort. Gene‐trap integration sites were mapped and processed as previously described 53.

### Flow cytometry

Cells were washed in PBS and, if necessary, detached by trypsinization. Routinely, 5 × 10^5^ cells were incubated for 30 min at 4°C in 100 μL PBS with the indicated fluorochrome‐conjugated antibody or stained sequentially with primary and fluorochrome‐conjugated secondary antibodies. Following fixation with 4% paraformaldehyde (PFA), cells were analysed on a Fortessa (BD). Data were analysed with the FlowJo software.

### CRISPR/Cas9‐mediated gene disruption

Cells stably expressing Cas9 and WDR81 or WDR91 specific sgRNAs were created by cotransfection of Cas9, sgRNA plasmids and packaging plasmids in 293ET, following the same protocol used for the generation of stable cell lines. WDR81 depletions by transient Cas9 and sgRNA expression were generated by cotransfecting HeLa cells with the Cas9 and WDR81 sgRNA#1 plasmids using TransIT‐HeLaMONSTER (Mirus) according to the manufacturer's instructions. After 24 h of transfection, cells were enriched for 72 h by puromycin and hygromycin selection and then single cell cloned. WDR81 knockout clones were identified using Immunoblot.

### Immunoblotting

Cells were washed in PBS and, if necessary, detached by trypsinization and lysed in 1% Triton X‐100 in TBS with 0.5 mm phenylmethylsulfonyl fluoride (PMSF), 10 mm iodoacetamide (IAA), and Roche complete protease inhibitor for 30 min on ice. Post‐nuclear supernatants were heated at 40°C for 20 min in SDS sample buffer, separated by SDS‐PAGE, and proteins transferred to PVDF membranes (Milipore). Membranes were blocked in 5% milk (Marvel) in PBS/0.2% Tween and probed with the indicated antibodies. Reactive bands were visualized using SuperSignal West Pico chemiluminescent substrate (Thermo Fisher Scientific). To quantitate protein levels, X‐ray films were scanned and subjected to densitometric analysis using ImageJ. Signal was then normalized by exposure and loading control prior to plotting on bar charts. Error bars represent the SEM for three technical repeats. Statistical significance was evaluated by paired, two‐tailed *t*‐tests.

### Confocal microscopy

Cells were grown overnight on round 13 mm glass coverslips, fixed in 4% PFA and permeabilized with 0.5% Triton X‐100, each step followed by two PBS washes. Where indicated, HeLa cytosol was extracted by incubating cells for 40 second in PBS containing 0.05% saponin and then immediately formaldehyde fixed. Cells were then blocked for 30 min with 5% FCS in PBS and incubated with primary antibody for 45–60 min at room temperature. Following three washes in blocking buffer, fluorescently coupled secondary antibody was applied for 30 min. After three washes in blocking buffer and three washes in water, coverslips were mounted in ProLong Gold Antifade Mountant with DAPI (Thermo Fisher Scientific) and imaged on a Zeiss LSM880 Confocal Microscope. Images were processed using the Zen 2011 software (Zeiss).

### EGFR degradation assay

For quantification by immunoblot, cells were incubated in HBSS amino acid depleted media (Thermo Fisher Scientific, 24020117) with 10 µg/mL cycloheximide and, where indicated, with 400 nm bafilomycin A (Sigma‐Aldrich) for 1 h prior to continuous stimulation with EGF (100 ng/mL) for the indicated times. Cells were then lysed and processed as described above. Three experimental repeats were subjected to densitometric analysis using the ImageJ software. The resulting plot was adjusted to reflect mean percentage of normalized pEGFR levels at 45 min ± SEM.

For visualization of endocytosed EGFR by confocal microscopy, cells seeded on cover slips were amino acid starved for 1 h prior to a 5 min incubation with EGF‐Alexa‐555 (Thermo Fisher Scientific, E‐35350) on ice. Cells were then washed twice in cold PBS and incubated at 37°C for the indicated times. Cells were then fixed in 4% PFA, washed, EEA1 stained and mounted. Single confocal images were acquired as described above (three independent experiments with ≥5 fields each per condition, average of two cells per field, randomly selected). The degree of colocalization between EGF‐Alexa‐555 and EEA1 was measured by Manders M1/M2 colocalization coefficients using the ZEN 2011 software (Zeiss) and normalized to the 20 min time point. Peak colocalization between EEA1 and EGFR was previously reported at 15 min 84, 85. In this study, we incubated a further 5 min at 37°C (total 20 min) to allow cells to equilibrate after pulsing on ice.

### Dextran, Magic Red and Lysosensor labelling

To label the endocytic pathway with dextran, cells were loaded for 16 h with 1 mg/mL dextran Alexa Fluor 488 10 000 MW, anionic (Thermo Fisher Scientific, D‐22910), washed, fixed, permeabilized and stained for intracellular tetherin as described above. To label endosomal/lysomal compartments containing the active acid hydrolase cathepsin B, live cells on coverslips were incubated for 5 min at 37°C with Magic Red MR‐(RR)_2_ Cathepsin B substrate (1:2600 dilution) (ImmunoChemistry Technologies, 938) and then imaged on an incubated Zeiss LSM710 Confocal Microscope. To assess acidic organelles, cells were labelled with LysoSensor Yellow/Blue DND‐160 (Thermo Fisher Scientific, L‐7545), a ratiometric probe that produces blue fluorescence in a neutral environment but shifts to yellow fluorescence in more acidic compartments (pKa ≈ 4.2). Cells were incubated for 1 min at 37°C with LysoSensor and then imaged on an incubated Zeiss LSM710 Confocal Microscope. Cells were excited at 405 nm and images were taken at 455.5 ± 32.5 and 570.5 ± 70.5 nm of emission. Zen 2011 (Zeiss) and ImageJ software were used for the analysis and processing of confocal images. The graph shows the mean value with the standard error mean of the yellow/blue ratios obtained from three experimental repeats (20 cells spread across 4 confocal fields for each experimental condition).

### Delivery of endocytosed dextran to lysosomes

A previously described protocol was used to measure the delivery of endocytosed dextran to lysosomes by quantitative live cell confocal microscopy 61. Briefly, HeLa cells were loaded for 2 h with 1 mg/mL Dextran Alexa Fluor 488 10 000 MW, anionic, fixable (Thermo Fisher Scientific) and then chased for 1 h in dextran‐free IMDM. Cells were then washed in PBS and incubated with Magic Red MR‐(RR)2 Cathepsin B substrate (1:2600 dilution) (ImmunoChemistry Technologies). Single confocal images were acquired using an incubated Zeiss LSM710 Confocal Microscope (three independent experiments with ≥5 fields each per condition, average of 1–2 cells per field, randomly selected). The degree of colocalization between Dextran Alexa Fluor 488 and Magic Red was measured by Manders M1/M2 colocalization coefficients using the ZEN 2011 software (Zeiss).

### Immunoprecipitation and mass spectrometry

Cells were lysed in 1% Triton X‐100 or 1% NP‐40 in TBS with 0.5 mm PMSF, 10 mm IAA and Roche complete protease inhibitor for 30 min on ice. Post‐nuclear supernatants were pre‐cleared with protein A and IgG‐Sepharose (Sigma‐Aldrich) and incubated with EZview Red Anti‐HA Affinity Gel (Sigma‐Aldrich) or anti‐GFP (Roche, 11814460001) plus protein A sepharose for 4 h at 4°C. Following one wash in 1% and four washes in 0.5% lysis buffer, immunoprecipitated proteins were eluted in Lamelli buffer. Samples were then resolved by SDS‐PAGE, gel lanes were excised and proteins were digested in‐gel. Tryptic peptides were analysed by LCMS/MS and the raw files processed in Proteome Discoverer 1.4. Data were searched using Mascot against the Human Uniprot database including common contaminants and a randomised reverse database. Results were filtered to a PSM FDR of 1%.

## Supporting information


**Figure S1. Haploid genetic screen is validated by the identification of components of the SCF^βTrCP2^ E3 ubiquitin ligase complex, known to be required for Vpu‐mediated downregulation of CD4 and degradation of CD4 and tetherin.** A) Schematic view of the Vpu IRES CFP construct. B) Schematic of sorting gates designed to avoid selection of rare cells that have lost Vpu expression (shown inside the yellow dashed oval). C) Bubble plot illustrating the hits from the CD4^high^ screen. Bubble size is proportional to the number of independent inactivating gene‐trap integrations identified (shown in brackets). D–E) Cytofluorometric analysis of tetherin and CD4 in control, Vpu expressing and Vpu expressing βTrCP2/1 depleted KBM7s (D) and CEMT4 (E) cells.
**Figure S2: Cell surface tetherin levels are partially rescued by WDR81 depletion in CEMT4 and HeLa cells.** A) Cytofluorometric analysis of cell surface tetherin expression in CEMT4 T cells: wild‐type (WT – blue dotted histogram), Vpu‐expressing (Vpu – red dotted histogram) and Vpu‐expressing CEMT4 cells depleted of WDR81 (KO – orange shaded histograms) by stable expression of two independent sgRNAs. Number in black represents median fluorescence signal. B) Median plasma membrane tetherin signal from three independent experiments using cells in ‘A’ was plotted ± SEM. * p ≤ 0.05. C) Cytofluorometric analysis of cell surface tetherin expression in HeLa cells: wild‐type (WT – blue dotted histogram), Vpu expressing (Vpu – red dotted histogram) and Vpu‐expressing HeLa cells depleted of WDR81 (KO – orange shaded histogram). Number in black represents median fluorescence signal. D) Immunostaining of tetherin in wild‐type and Vpu‐expressing HeLa cells. Showing the same images as in Figure 2D (left panels) with increased contrast in the tetherin channel. Processed with ZEN 2011. Scale bars represent 10 µm.
**Figure S3: Depletion of WDR81 does not impede endolysosomal acidification. Tetherin accumulates in early EEA1 positive but not in late LAMP1 positive compartments.** A) Control and WDR81KO cells were labelled with LysoSensor Yellow/Blue DND‐160, a ratiometric probe that produces blue fluorescence in a neutral environment (green) but shifts to yellow fluorescence in more acidic compartments (red). B) Averages of yellow/blue ratios obtained from three experimental repeats (20 cells spread across 4 confocal fields for each experimental condition) were plotted ± SEM. **p ≤ 0.01. C) Immunostaining of tetherin and EEA1 in control and WDR81KO cells. D) Immunostaining of tetherin and LAMP1 in control and WDR81KO cells. All scale bars represent 5 µm.
**Figure S4: WDR81 depletion delays EGFR degradation.** Confocal microscopy of control and WDR81KO HeLa cells stimulated with EGF‐Alexa 555 and chased for the indicated times. Detector gain was decreased 1.25‐fold for the 20, 60 and 180 min time points (as compared to 0 min). Scale bars represent 10 µm.
**Figure S5: C‐terminal GFP fusion rendered an exogenous WDR81 construct non‐functional and unable to bind WDR91.** A) Immunostaining of tetherin in WDR81‐GFP complemented WDR81KO cells. Scale bars represent 10 µm. B) Mass spectrometric analysis of WDR81‐GFP immunoprecipitates. Wild‐type HeLa cells were used as control.
