# Supplementary material for: A Genetic Screen Identifies a Critical Role for the WDR81‐WDR91 Complex in the Trafficking and Degradation of Tetherin
Source: Traffic. 2016 May 25;17(8):940–58. doi: 10.1111/tra.12409 (PMC5025723; doi:10.1111/tra.12409)
Supplement: Supplementary file 1 — Figure S1. Haploid genetic screen is validated by the identification of components of the SCFβTrCP2 E3 ubiquitin ligase complex, known to be required for Vpu‐mediated downregulation of CD4 and degradation of CD4 and tetherin. A) Schematic view of the Vpu IRES CFP construct. B) Schematic of sorting gates designed to avoid selection of rare cells that have lost Vpu expression (shown inside the yellow dashed oval). C) Bubble plot illustrating the hits from the CD4high screen. Bubble size is proportional to the number of independent inactivating gene‐trap integrations identified (shown in brackets). D–E) Cytofluorometric analysis of tetherin and CD4 in control, Vpu expressing and Vpu expressing βTrCP2/1 depleted KBM7s (D) and CEMT4 (E) cells. Figure S2: Cell surface tetherin levels are partially rescued by WDR81 depletion in CEMT4 and HeLa cells. A) Cytofluorometric analysis of cell surface tetherin expression in CEMT4 T cells: wild‐type (WT – blue dotted histogram), Vpu‐expressing (Vpu – red dotted histogram) and Vpu‐expressing CEMT4 cells depleted of WDR81 (KO – orange shaded histograms) by stable expression of two independent sgRNAs. Number in black represents median fluorescence signal. B) Median plasma membrane tetherin signal from three independent experiments using cells in ‘A’ was plotted ± SEM. * p ≤ 0.05. C) Cytofluorometric analysis of cell surface tetherin expression in HeLa cells: wild‐type (WT – blue dotted histogram), Vpu expressing (Vpu – red dotted histogram) and Vpu‐expressing HeLa cells depleted of WDR81 (KO – orange shaded histogram). Number in black represents median fluorescence signal. D) Immunostaining of tetherin in wild‐type and Vpu‐expressing HeLa cells. Showing the same images as in Figure 2D (left panels) with increased contrast in the tetherin channel. Processed with ZEN 2011. Scale bars represent 10 µm. Figure S3: Depletion of WDR81 does not impede endolysosomal acidification. Tetherin accumulates in early EEA1 positive but not in late LAM [file TRA-17-940-s001.pdf]

Supporting information:

A genetic screen identifies a critical role for the WDR81-WDR91 complex in the trafficking and degradation of tetherin

Radu Rapiteanu[1], Luther J. Davis[2], James C. Williamson[1], Richard T. Timms[1], J. Paul Luzio[2] and Paul J. Lehner[1*]

Cambridge Institute for Medical Research and [1]Departments of Medicine and [2]Clinical Biochemistry, University of Cambridge School of Clinical Medicine, Wellcome Trust/MRC Building Biomedical Campus, Hills Road, Cambridge, CB2 0XY, UK

[*]Corresponding author: Paul J. Lehner, pjl30@cam.ac.uk

Figure S1

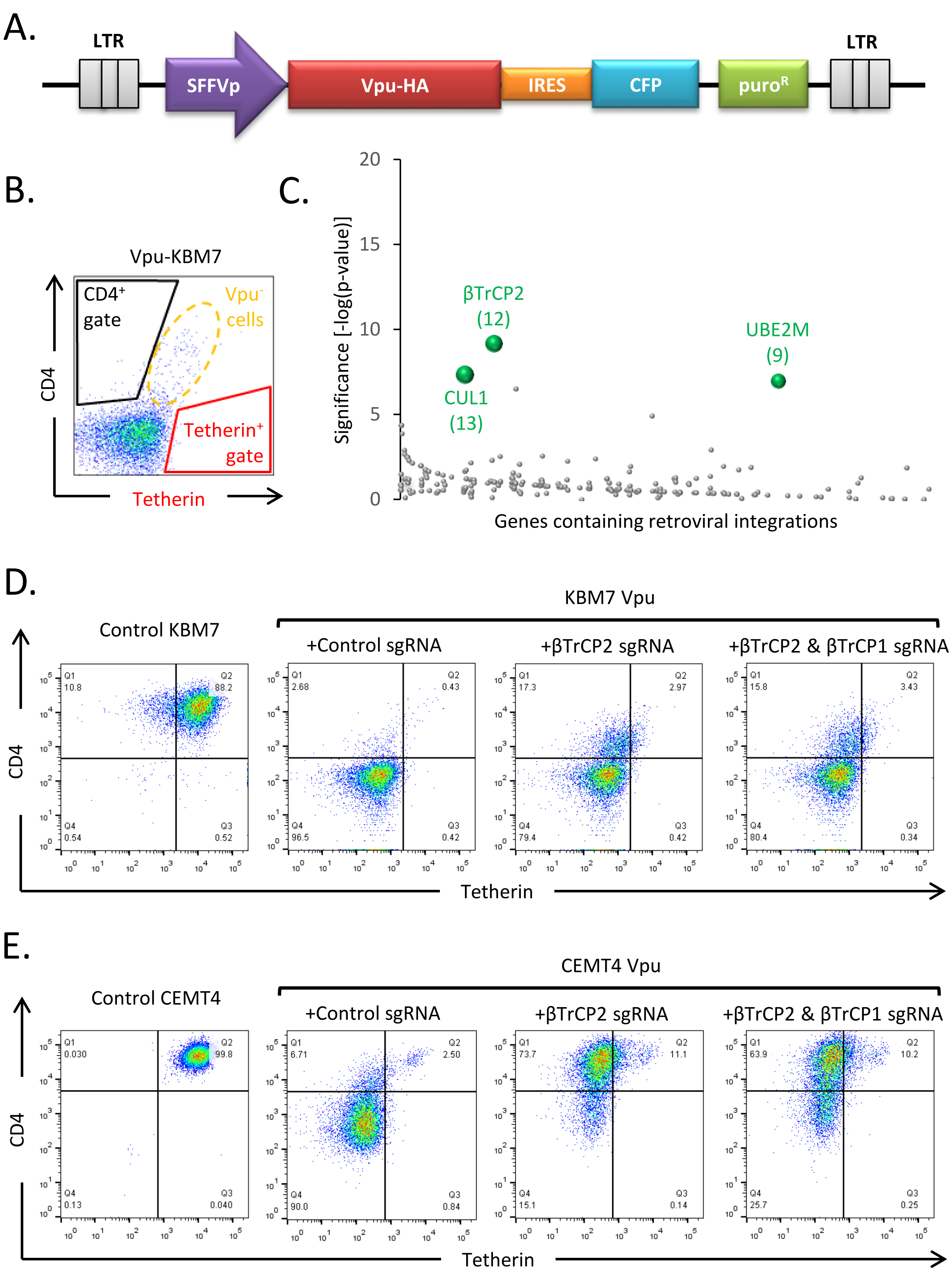

Figure S1: Haploid genetic screen is validated by the identification of components of the SCF$^{\beta TrCP2}$ E3 ubiquitin ligase complex, known to be required for Vpu-mediated downregulation of CD4 and degradation of CD4 and tetherin. A) Schematic view of the Vpu IRES CFP construct. B) Schematic of sorting gates designed to avoid selection of rare cells that have lost Vpu expression (shown inside the yellow dashed oval). C) Bubble plot illustrating the hits from the CD4^{high} screen. Bubble size is proportional to the number of independent inactivating gene-trap integrations identified (shown in brackets). D-E) Cytofluorometric analysis of tetherin and CD4 in control, Vpu expressing and Vpu expressing βTrCP2/1 depleted KBM7s (D) and CEMT4 (E) cells.

Figure S2

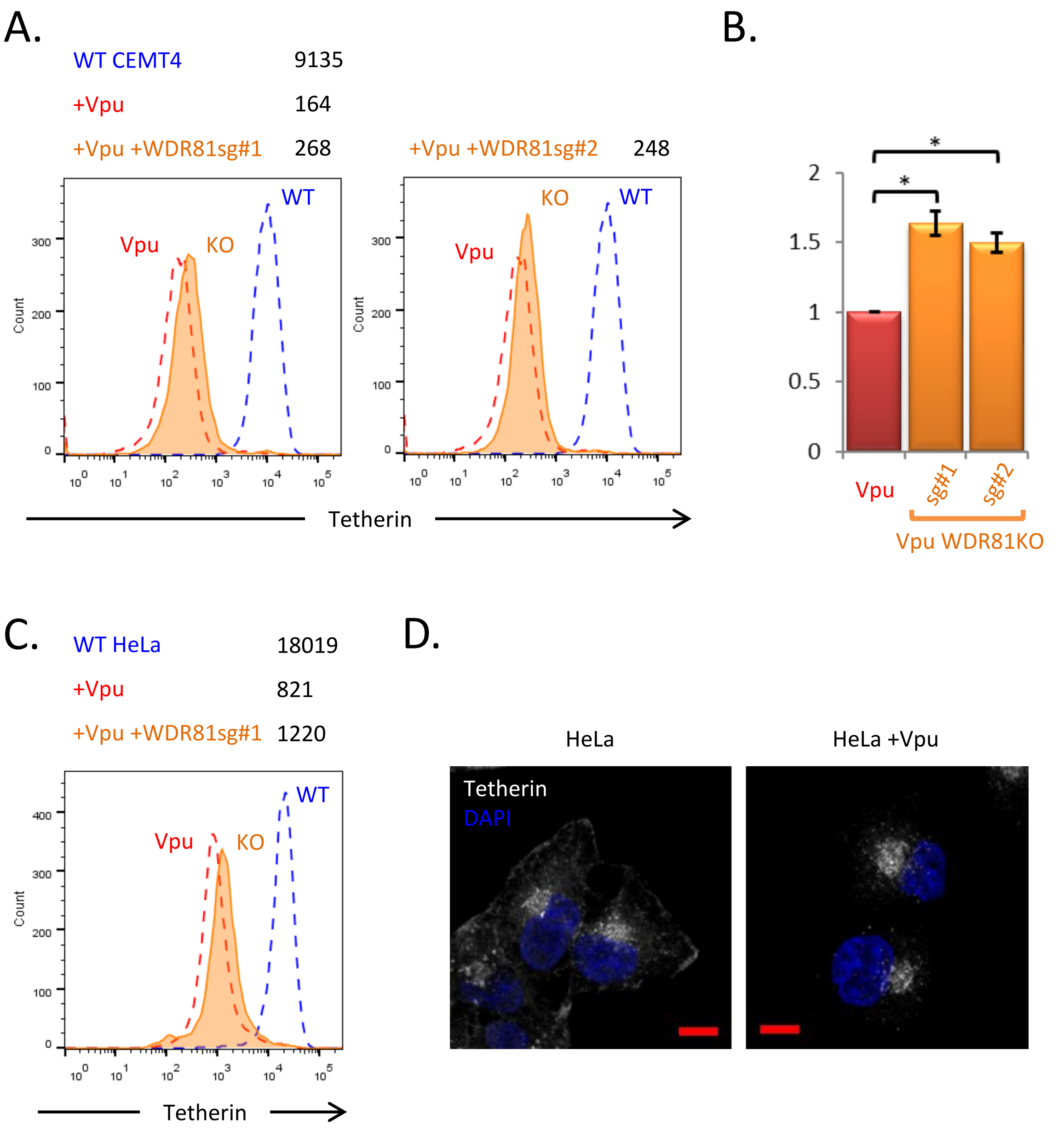

Figure S2: Cell surface Tetherin levels are partially rescued by WDR81 depletion in CEMT4 and HeLa cells. A) Cytofluorometric analysis of cell surface tetherin expression in CEMT4 T cells: wildtype (WT - blue dotted histogram), Vpu-expressing (Vpu - red dotted histogram) and Vpu-expressing CEMT4 cells depleted of WDR81 (KO - orange shaded histograms) by stable expression of 2 independent sgRNAs. Number in black represents median fluorescence signal. B) Median plasma membrane tetherin signal from 3 independent experiments using cells in ‘A’ was plotted ± SEM. * P ≤ 0.05. C) Cytofluorometric analysis of cell surface tetherin expression in HeLa cells: wildtype (WT - blue dotted histogram), Vpu-expressing (Vpu - red dotted histogram) and Vpu-expressing HeLa cells depleted of WDR81 (KO - orange shaded histogram). Number in black represents median fluorescence signal. D) Immunostaining of tetherin in wildtype and Vpu-expressing HeLa cells. Showing the same images as in Figure 2D (left panels) with increased contrast in the tetherin channel. Processed with ZEN 2011. Scale bars represent 10µm.

Figure S3

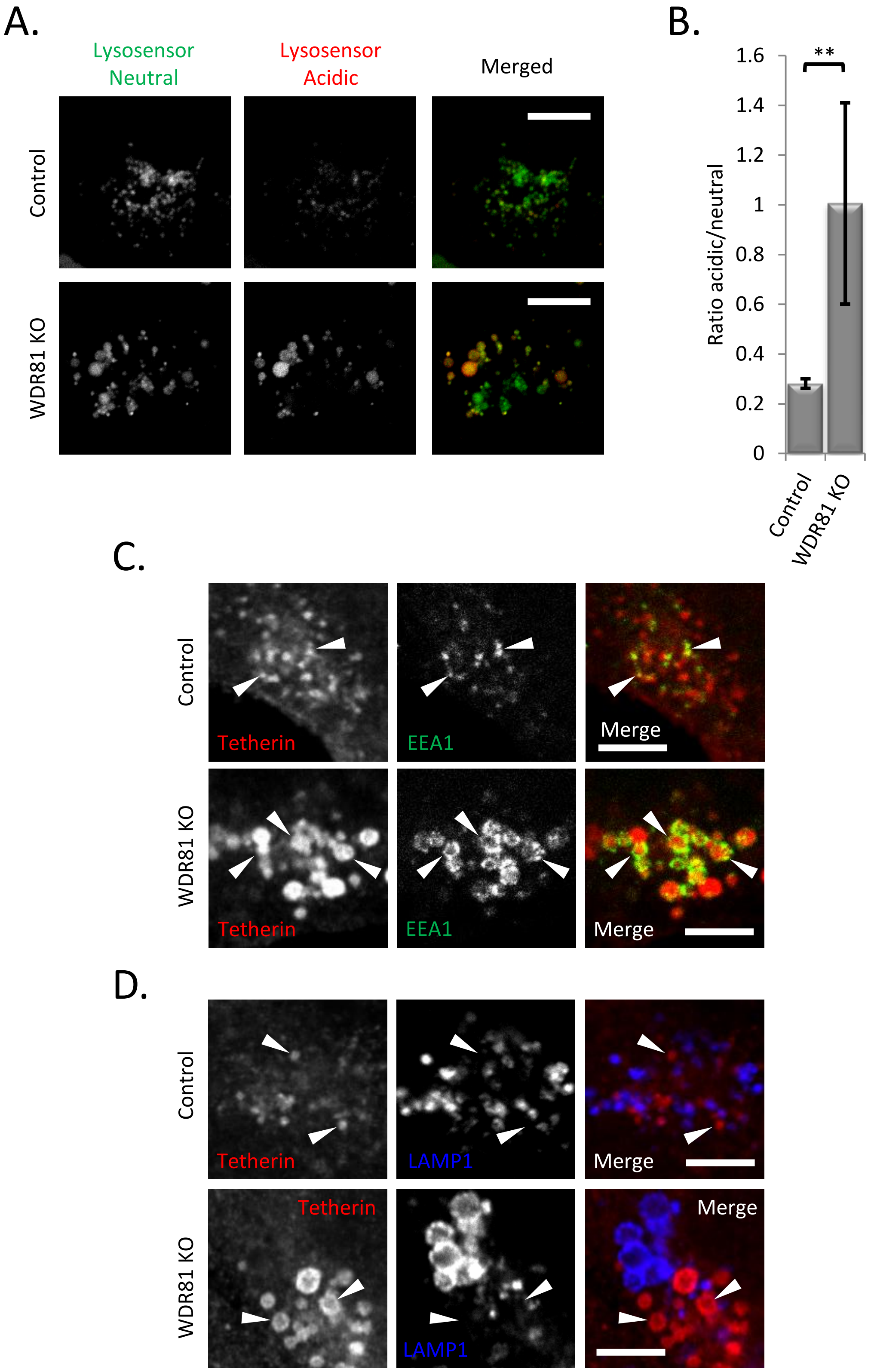

Figure S3: Depletion of WDR81 does not impede endolysosomal acidification. Tetherin accumulates in early EEA1 positive but not in late LAMP1 positive compartments. A) Control and WDR81KO cells were labelled with LysoSensor Yellow/Blue DND-160, a ratiometric probe that produces blue fluorescence in a neutral environment (green) but shifts to yellow fluorescence in more acidic compartments (red). B) Averages of yellow/blue ratios obtained from three experimental repeats (20 cells spread across 4 confocal fields for each experimental condition) were plotted ± SEM. ** $P \leq 0.01$. C) Immunostaining of tetherin and EEA1 in control and WDR81KO cells. D) Immunostaining of tetherin and LAMP1 in control and WDR81KO cells. All scale bars represent 5µm.

Figure S4

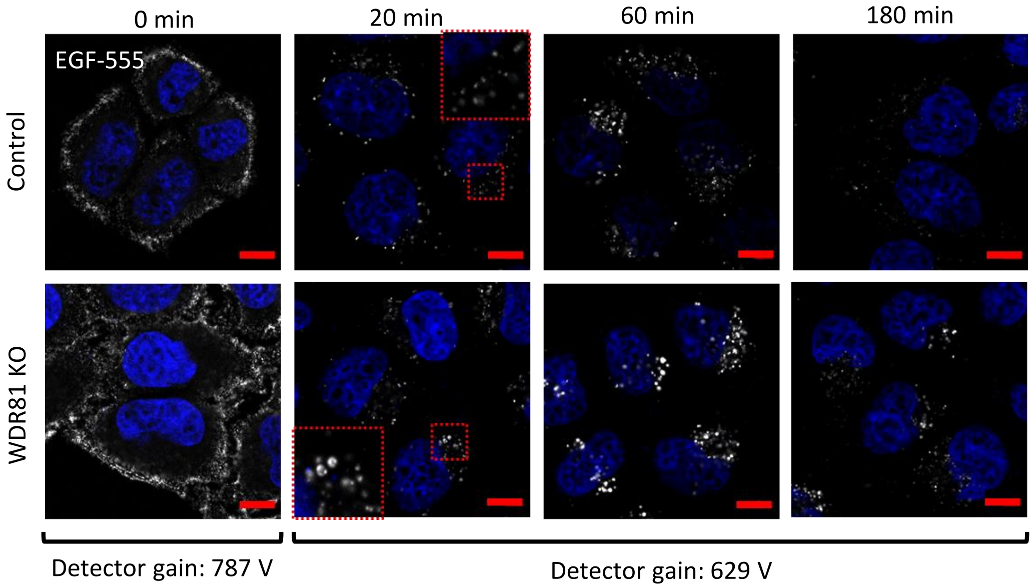

Figure S4: WDR81 depletion delays EGFR degradation. Confocal microscopy of control and WDR81KO HeLa cells stimulated with EGF-Alexa 555 for 5 min and chased for the indicated times. Detector gain was decreased 1.25 fold for the 20, 60 and 180 min time points (as compared to 0 min). Scale bars represent 10 μm.

Figure S5

A.

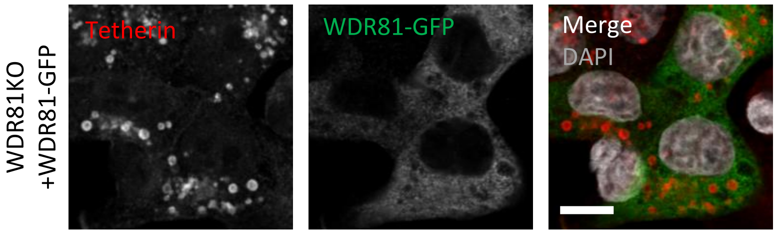

B.

Accession	Gene ID	IP: HA, Detergent: NP-40, Wild-type: Unique peptides	IP: HA, Detergent: NP-40, Wild-type: Coverage	IP: HA, Detergent: NP-40, WDR81 KO + 2xHA-WDR81: Unique peptides	IP: HA, Detergent: NP-40, WDR81 KO + 2xHA-WDR81: Coverage	IP: GFP, Detergent: NP-40, Wild-type: Unique peptides	IP: GFP, Detergent: NP-40, Wild-type: Coverage	IP: GFP, Detergent: NP-40, WDR81 KO + WDR81-GFP: Unique peptides	IP: GFP, Detergent: NP-40, WDR81 KO + WDR81-GFP: Coverage
Q562E7	WDR81	5	4%	105	73%	0	0%	50	35%
A4D1P6	WDR91	0	0%	35	64%	0	0%	0	0%

Figure S5: C-terminal GFP fusion rendered an exogenous WDR81 construct non-functional and unable to bind WDR91. A) Immunostaining of tetherin in WDR81-GFP complemented WDR81KO cells. Scale bars represent 10µm. B) Mass spectrometric analysis of WDR81-GFP immunoprecipitates. Wild-type HeLa cells were used as control.
